# Characterization and functional analysis of *AhGPAT9* gene involved in lipid synthesis in peanut (*Arachis hypogaea* L.)

**DOI:** 10.3389/fpls.2023.1144306

**Published:** 2023-02-10

**Authors:** Yue Shen, Yi Shen, Yonghui Liu, Yang Bai, Man Liang, Xuyao Zhang, Zhide Chen

**Affiliations:** ^1^ Institute of Industrial Crops, Jiangsu Academy of Agricultural Sciences, Nanjing, China; ^2^ Jiangsu Key Laboratory for the Research and Utilization of Plant Resources, Institute of Botany, Jiangsu Province and Chinese Academy of Sciences (Nanjing Botanical Garden Mem. Sun Yat-Sen), Nanjing, China

**Keywords:** *Arachis hypogaea*, *AhGPAT9*, evolution analysis, triacylglycerol, oil content, fatty acid

## Abstract

GPAT enzymes (glycerol-3-phosphate 1-O-acyltransferase, EC 2.3.1.15) catalyze the initial and rate-limiting step of plant glycerolipid biosynthesis for membrane homeostasis and lipid accumulation, yet little research has been done on peanuts. By reverse genetics and bioinformatics analyses, we have characterized an AhGPAT9 isozyme, of which the homologous product is isolated from cultivated peanut. QRT-PCR assay revealed a spatio-temporal expression pattern that the transcripts of *AhGPAT9* accumulating in various peanut tissues are highly expressed during seed development, followed by leaves. Green fluorescent protein tagging of *AhGPAT9* confirmed its subcellular accumulation in the endoplasmic reticulum. Compared with the wild type control, overexpressed *AhGPAT9* delayed the bolting stage of transgenic *Arabidopsis*, reduced the number of siliques, and increased the seed weight as well as seed area, suggesting the possibility of participating in plant growth and development. Meanwhile, the mean seed oil content from five overexpression lines increased by about 18.73%. The two lines with the largest increases in seed oil content showed a decrease in palmitic acid (C16:0) and eicosenic acid (C20:1) by 17.35% and 8.33%, respectively, and an increase in linolenic acid (C18:3) and eicosatrienoic acid (C20:3) by 14.91% and 15.94%, respectively. In addition, overexpressed *AhGPAT9* had no significant effect on leaf lipid content of transgenic plants. Taken together, these results suggest that *AhGPAT9* is critical for the biosynthesis of storage lipids, which contributes to the goal of modifying peanut seeds for improved oil content and fatty acid composition.

## Introduction

Peanut (*Arachis hypogaea* L.) or groundnut is an important self-pollinated legume crop widely cultivated around the world for edible oil, food and feed use. Peanut kernels are comprised of over 50% oil and about 30% protein, as well as many minerals and vitamins (Janila et al., 2013). The fatty acid (FA) composition of peanut is unique, in that the total unsaturated fatty acid content exceeds 80%. The variation of oleic (O) and linoleic (L) fatty acids represents the most important quality traits for evaluating stability and nutrition, and high O/L ratio can increase the shelf life (Jung et al., 2000). China’s annual peanut production ranks first in the world, and more than 50% of its total output is used for oil extraction, which plays an important strategic role in ensuring the safety of edible oil. However, constraints including limited acreage and the increasing demand for lipid consumption means that improvement of peanut oil content and quality remains the focus of current scientific work.

Triacylglycerol (TAG) represents the major storage reserve in oilseeds, usually esterified by 3 fatty acids and 1 glycerol. It can provide energy support for germinating plants during the non-autotrophic stage before photosynthesis, and is also involved in plant development and stress resistance (Graham, 2008; Zhang et al., 2009; Cui et al., 2016). Many studies have demonstrated the existence of several parallel TAG biosynthetic pathways in plants, including the acyl-CoA-dependent Kennedy pathway (*de novo* DAG/TAG synthesis), the acyl-CoA-independent PC pathway (PC-derived DAG/TAG synthesis), and the monoacylglycerol (MAG) pathway, showing that plant lipid synthesis involves a complex metabolic network with multiple regulatory pathways and genes (Tumaney et al., 2001; Shi and Cheng, 2009; Chen et al., 2015; Bates, 2016).

Our focus on glycerol-3-phosphate acyltransferase (GPAT) bridges the two compartmentalization pathways of fatty acid synthesis in plastid and glycerolipid synthesis in the endoplasmic reticulum, and the first step of the acylation reaction catalyzed by it is considered to be the key rate-limiting step of the Kennedy pathway. As the carbon chain skeleton for TAG synthesis, glycerol-3-phosphate (G3P) harbors three fatty acid binding sites, *sn*-1, *sn*-2 and *sn*-3. GPATs mainly transfer the acyl groups on acyl-CoA to the *sn*-1 or *sn*-2 position hydroxyl groups of G3P to produce lysophosphatidic acid (LPA), a crucial intermediate for the formation of several acyl-lipids. In plants, different subcellular localization of GPATs may determine the metabolites of LPAs, such as extracellular lipid polyesters, membrane and storage lipids (Lee et al., 2016).

In *Arabidopsis*, ten GPAT homologs have been identified, of which *GPAT1~8* are the land-plant-specific *sn*-2-GPAT that is mainly involved in the biosynthesis of lipid polyesters such as cutin and suberin, and is associated with plant flower development and stress response (Li et al., 2007; Li-Beisson et al., 2009; Chen et al., 2011; Yang et al., 2012). Plastid-localized *ATS1* also can catalyze the acylation reaction at the *sn*-1 site of G3P using acyl-ACP as an acyl donor, and may be related to the mechanism of plant cold tolerance response (Payá-Milans et al., 2015; Sun et al., 2015). Loss of function of *GPAT1* located in mitochondria alters the fatty acid composition in floral tissues and seeds, but its effect on seed oil content is still controversial (Zheng et al., 2003; Bai et al., 2021). Undoubtedly, *Arabidopsis GPAT9*, which is similar to mammalian *GPAT3/4* function in fat synthesis, can be directly involved in the membrane lipids and TAG biosynthesis for plants as the *sn*-1 bifunctional enzyme gene localized in endoplasmic reticulum (Gidda et al., 2009; Shockey et al., 2016; Singer et al., 2016). In addition, *GPATs* involved in TAG accumulation have been cloned in *Lepidium latifolium* (Mohan et al., 2013), *Brassica napus* (Chen et al., 2014; Liu et al., 2015), *Helianthus annuus* (Payá-Milans et al., 2016), *Jatropha curcas* (Misra et al., 2017), *Physcomitrella patens* (Yang et al., 2019) and other plants.

In this study, a homologous transcript of *Arabidopsis GPAT9* gene (At5g60620), *AhGPAT9*, isolated from cultivated peanut, which encodes a fragment of 1131bp in length. The Conserved Domain Databases (CDD) was used to predict that its encoded protein possessed typical glycerol acyltransferase activity. The expression characteristics of *AhGPAT9* were analyzed to elucidate its biological function involved in plant lipid synthesis.

## Materials and methods

### Plant materials and growth conditions

Cultivated peanut (*Arachis hypogaea* cv. Tifrunner) seeds were sterilized in 2% (v/v) sodium hypochlorite for 10 min, rinsed thoroughly with deionized water, sown into nutrient-enriched soil, and grown in the phytotron at 25°C with a 16:8 h light: dark (L:D) photoperiod. *A. thaliana* ecotype Col-0 seeds were pre-grown in MS basal medium (Murashige and Skoog, 1962) for one week, and then transferred to nutrient-enriched soil in a growth chamber at 23°C with a 14:10 h L:D photoperiod. Hoagland’s nutrient solution was used for water and fertilizer management.

### Data mining of peanut GPATs

The sequence data of GPAT conserved domain (PF01553) was downloaded from Pfam database (http://pfam.xfam.org/), and the protein library of cultivated peanut and its two diploid progenitors (*Arachis duranensis* and *Arachis ipaensis*) were downloaded from PeanutBase database (https://www.peanutbase.org). The hidden Markov model was constructed by HMMER3.0 software to obtain the preliminary screened ID information of peanut GPAT. The protein sequences containing PF01553 domain were anchored and extracted by SeqHunter1.0 software, and the integrity of conserved domain of above sequences was analyzed by Pfam and SMART databases, while repetitive sequences and redundant transcripts were removed to screen the candidate genes of peanut *GPATs*.

### Phylogenetic and chromosome mapping analyses of peanut GPATs

*A. thaliana* GPAT sequences was retrieved from the Arabidopsis Information Resource (https://www.arabidopsis.org). The multiple sequence alignment analysis of GPAT proteins from *Arachis* and *Arabidopsis* were analyzed by Clustal-X software, then phylogenetic tree was constructed by maximum likelihood method with 1000 bootstraps using MEGA-X software. The genome sequence and genome structure annotation data of cultivated peanut were downloaded, from which the chromosomal location data of *AhGPATs* were extracted, and physical location diagram of candidate genes on peanut chromosome were drawn by TBtools software (Chen et al., 2020).

### Cloning of *AhGPAT9*

The coding sequence of *AhGPAT9* (1131bp) was amplified from reverse-transcribed RNA isolated from peanut seedlings, the sequence data with the database locus number Arahy.5QGNM was obtained from PeanutBase database. Primers were designed by PrimerPrimer5.0 software, PCR amplification was performed using KOD-Plus-Neo high-fidelity enzyme (TOYOBO, Shanghai, China), and the clone was ligated into the pEASY vector (TransGen, Beijing, China). The primers used for amplification of CDS fragment are listed in **Supplementary Table S1**.

### Bioinformatics analysis

We chose the CDD database (https://www.ncbi.nlm.nih.gov/cdd) for initial identification of conserved domain in AhGPAT9 protein sequence. Then the prediction analyses of physicochemical parameters, hydrophobicity, secondary structure, transmembrane helices, signal peptides, functional domains and phosphorylation sites of the protein encoded by *AhGPAT9* were performed by ProtParam (https://web.expasy.org/protparam), ProtScale (https://web.expasy.org/protscale), SOPMA (https://npsa-prabi.ibcp.fr/cgi-bin/npsa_automat.pl?page=/NPSA/npsa_sopma.html), TMHMM (https://services.healthtech.dtu.dk/service.php?TMHMM), SignalIP (https://services.healthtech.dtu.dk/service.php?SignalP), SMART (http://smart.embl-heidelberg.de) and NetPhos (https://services.healthtech.dtu.dk/service.php?NetPhos) databases, respectively. Phylogenetic analysis of AhGPAT9 homologs from different species was performed by screening homologous sequences through the BLASTp non-redundant protein database (https://blast.ncbi.nlm.nih.gov), and then constructing a phylogenetic tree by maximum likelihood method with 1000 bootstraps using MEGA-X software.

### RNA isolation and QRT-PCR analysis

Total RNA was extracted from various tissues of peanut with plant RNA isolation kit (Sangon, Shanghai, China) following the manufacturer’s instructions. First-strand cDNA was synthesized using PrimeScript™ RT reagent Kit with gDNA eraser (TaKaRa, Dalian, China). PCR amplification was performed using SYBR Premix Ex Taq™ kit (TaKaRa, Dalian, China) with *AhACT11* (Xu et al., 2021) as the internal reference, each template was repeated three times, and the data were analyzed by comparative cycle threshold method (2^-ΔΔCT^). Quantitative real-time (qRT) PCR assay was performed in 20μl reaction volumes using a QuantStudio5 real-time PCR system (Applied Biosystems, California, USA). The primers used for qRT-PCR are listed in **Supplementary Table S1**.

### Subcellular localization assay

Prediction analysis of the subcellular location of *AhGPAT9* was performed by ProtCompVersion9.0 (http://linux1.softberry.com/berry.phtml?topic=protcomppl&group=programs&subgroup=proloc) software. Meanwhile, *AhGPAT9* and *HDEL* coding sequence (excluding the stop codon) were amplified and fused with *EGFP* and *mCherry* reporter genes, respectively, at the C-terminal in the pSuper1300^+^ vector. The recombinant plasmids *AhGPAT9-EGFP* and *HDEL-mCherry* were transferred into *Agrobacterium tumefaciens* strain *GV3101* by freeze-thaw method. Then tobacco leaves were transfected by infiltration using injection method, and incubated for 2-3 d under normal cultural conditions before performing fluorescence assays using an UltraVIEW VoX laser confocal imaging analysis system (PerkinElmer, Massachusetts, USA). The primers used for subcellular location are listed in **Supplementary Table S1**.

### Generation of transgenic *Arabidopsis* expressing *AhGPAT9*

The coding sequence of *AhGPAT9* was built into the modified binary vector pSuper1300^+^ containing the *Super* promoter (Shen et al., 2015). Then the recombinant plasmid was transferred into *Agrobacterium tumefaciens* strain *GV3101* to infect inflorescences of *Arabidopsis* using the floral-dipping method. Mendelian inheritance of hygromycin resistance was showed as single dominant locus in T1 and T2 generation of all transgenic plants, and the homozygous lines of T3 generation were used for phenotype analysis and determination of oil content and FA composition. The primers are used for overexpression are listed in **Supplementary Table S1**.

### Lipid extraction and analysis

The extraction of seed oil (Li et al., 2006) and total leaf lipid (Kim et al., 2015) was extracted using micro-extraction method, 10-20 mg freeze-dried samples were taken and repeated five times for each treatment. TAG component of total leaf lipid was separated using thin-layer chromatography method (TLC). Fatty acid composition of TAG in seeds and leaves was quantitatively analyzed by internal standard method using TAG-17:0 for normalization. The methyl esterification products were determined using gas chromatography method with Thermo TRACE GC (ThermoFisher, Massachusetts, USA), and 37 fatty acid methyl ester mixture (Sigma, Missouri, USA) was used as the standard substance. According to the peak time of different fatty acids in the standard, the fatty acid components of each sample were analyzed, and then the relative molar content of each fatty acid was calculated based on the peak area.

## Results

### Identification and chromosomal localization of peanut GPATs

**Figure 1:**
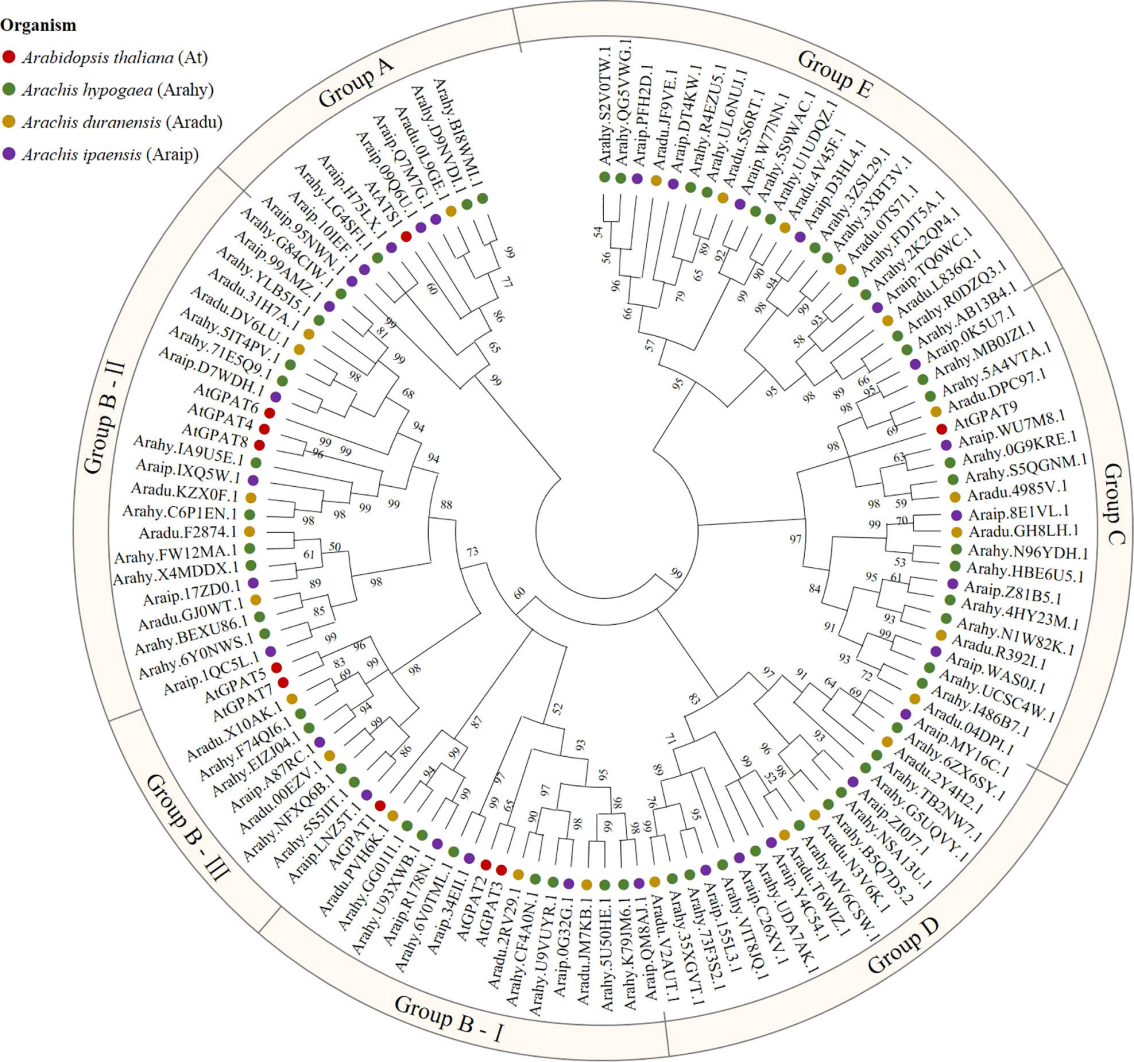
Phylogenetic analysis of glycerol-3-phosphate acyltransferase (GPAT) family in peanut. GPATs in cultivated peanut (*Arachis hypogaea*) and its two diploid progenitors (*Arachis duranensis* and *Arachis ipaensis*), as well as *A. thaliana* were used to construct a phylogenetic tree by maximum likelihood method with 1000 bootstraps using MEGA software (version X). Red = *A. thaliana*; Green = *Arachis hypogaea*; Yellow = *Arachis duranensis*; Purple = *Arachis ipaensis*.

**Figure 2:**
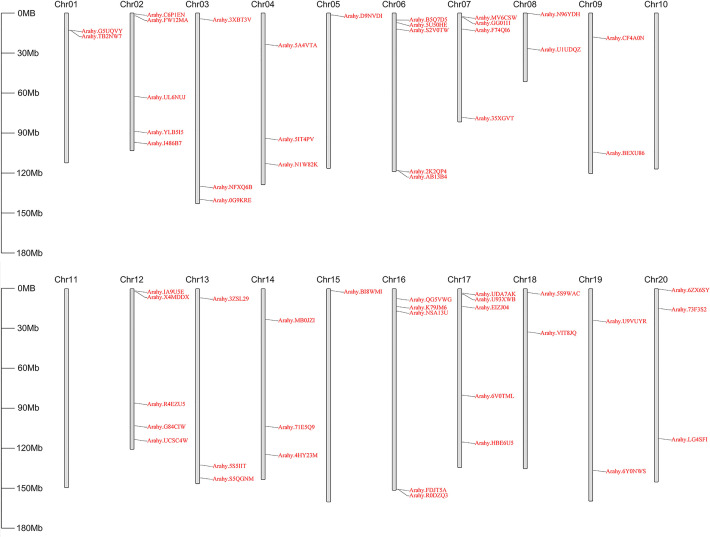
Physical location of *AhGPATs* genes on peanut chromosomes. The 56 predicted *AhGPATs* genes from cultivated peanut (*Arachis hypogaea*) were used to construct a physical location distribution map on chromosomes using TBtools software. Chromosome numbers are indicated on the top of each scaffold. Chromosome size is shown on the left vertical scale.

To identify peanut *GPAT* genes and their homologs, the PlsC acyltransferase domain of Pfam (PF01553) was used as a probe to screen 56, 25 and 31 proteins, respectively, with complete domain motifs from the protein sequence data of cultivated peanut and its two diploid progenitors. Based on the ploidy relationship among them, it was found that the quantity of GPATs in cultivated peanut was consistent with the sum of GPAT members in wild peanut. Phylogenetic analysis of GPAT proteins in peanut and *Arabidopsis* was performed by maximum likelihood method using MEGA software, and the results showed that they were divided into five subfamilies. As shown in **Figure 1**, sequences homologous to AtATS1 and AtGPAT9 constituted A and C independent groups, respectively, with *sn*-1 acyltransferase properties (Nishida et al., 1993; Singer et al., 2016). Group B contained all the land-plant-specific *sn*-2-CPAT, which mainly performed the function of a membrane-bound protein and could be further classified into three distinct conserved sub-clades based on the key stages of the morphological and functional evolution: cutin synthesis (II), suberin synthesis (III) and other vascular ancient branches (I) (Yang et al., 2012). In addition, there was no clustering of GPAT for *A. thaliana* within group D and group E, which suggests that they might be glycerol-3-phosphate acyltransferases specific to peanut. The 56 *AhGPAT*s members were found to be unevenly distributed across 18 chromosomes of cultivated peanut genome except for chromosomes 10 and 11 (**Figure 2**). Chromosomes 02, 06, 12, 16 and 17 contained five genes, chromosome 7 contained four genes, chromosomes 03, 04, 13, 14 and 20 contained three genes, chromosomes 01, 08, 09, 18 and 19 contained two genes, and chromosomes 05 and 15 contained only one gene.

### Cloning and bioinformatics analysis of *AhGPAT9*

In this study, a fragment (**Figure S1**) homologous to *AtGPAT9* for *Arabidopsis* was obtained by PCR amplification. After sequencing and alignment, it was found that this gene was consistent with the *Arahy.5QGNM* reference sequence, named *AhGPAT9* (Genebank accession number: MN124513) and located on chromosome 13 in peanut (**Figure 2**). The length of the coding sequence for *AhGPAT9* was 1131bp, which encoded 376 amino acids. Physicochemical property analysis showed that AhGPAT9 was an unstable protein with molecular formula C_1983_H_3083_N_533_O_529_S_21_, molecular weight 43.528kD, isoelectric point 9.09, fat coefficient 91.97 and stability coefficient 46.56. Meanwhile, the total average hydrophobicity coefficient was -0.110, making it a hydrophilic protein (**Figure S2A**). Secondary structure prediction results showed that α-helix accounted for 48.40%, β-turn accounted for 3.46%, random coil accounted for 33.24%, and extended strand accounted for 14.89%, indicating that α-helix and random coil are the main components of the secondary structure for AhGPAT9 (**Figure S2B**). Phosphorylation site prediction results showed that the protein had 25 phosphorylation sites, including 5 threonine, 17 serine and 3 tyrosine (**Figure S2C**), suggesting that phosphorylation modification may be involved in regulating the activity of AhGPAT9 protein, and are dominated by the serine sites. In addition, it was found that the protein contains no signal peptide but three transmembrane domains and a PlsC functional domain (**Figure S2D, E**), suggesting that AhGPAT9 may be associated with glycerolipid synthesis given the phosphor-acyltransferase activity of the PlsC domain.

**Figure 3:**
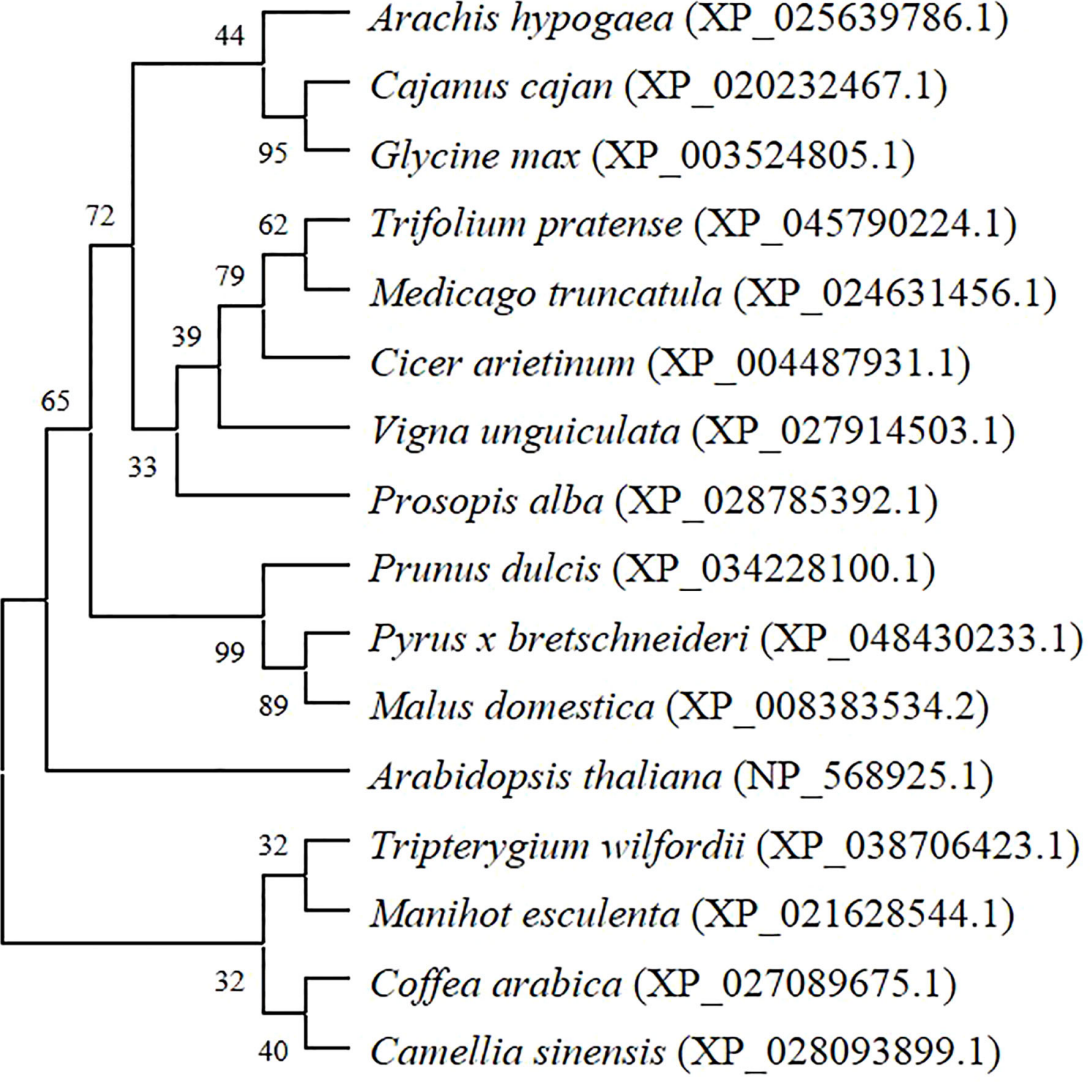
Phylogenetic tree of peanut AhGPAT9 proteins with related GPATs from other plant species. Species names are in italics, and accession numbers of reference sequences in NCBI are in parentheses.

Fourteen protein sequences with high similarity to AhGPAT9 were selected from the non-redundant protein database (sequence identity 84.51~88.82%). Protein functional annotation showed that they were GPAT9 proteins from different species. Multiple sequence alignment analysis revealed that all these protein sequences had a complete PlsC functional domain. Furthermore, the phylogenetic tree constructed by the maximum likelihood method of MEGA software (**Figure 3**), shows eight legume sources of GPATs clustered into the same group, of which peanut AhGPAT9 has the closest molecular distance to dhal CcGPAT9 (XP_020232467.1) and soybean GmGPAT9 (XP_003524805.1), suggesting that AhGPAT9 may be functionally similar to both of them.

### Expression pattern and subcellular localization of *AhGPAT9*

**Figure 4:**
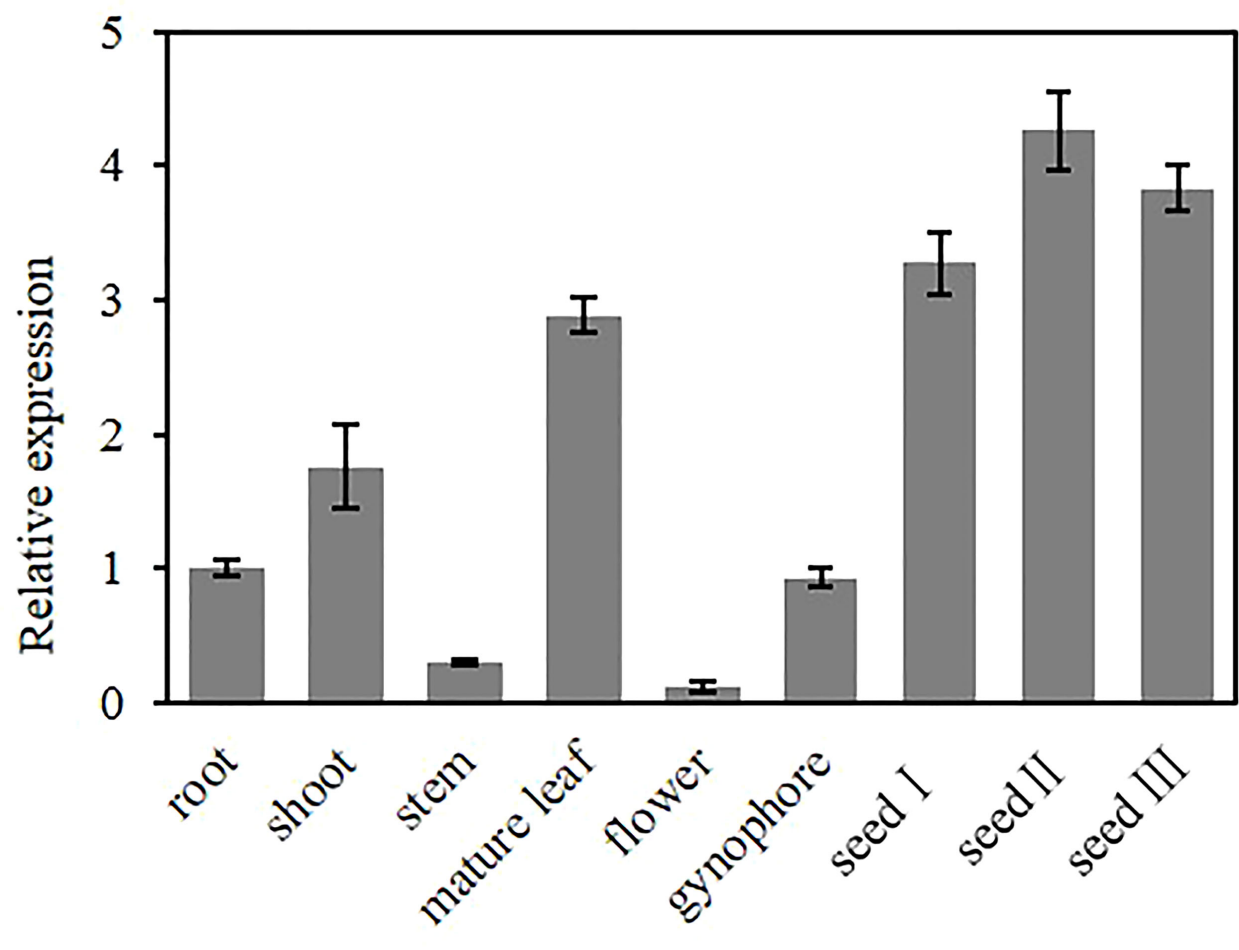
Expression analysis of *AhGPAT9* using qRT-PCR in diverse tissues of peanut. The root and shoot samples were taken at seedling stage, the stem, leaf, flower, gynophore and seed samples were taken at adult-plant stage. Seed І, seed II, and seed Ш represent the early, middle and late stages of seed development, respectively. Relative mRNA abundance was normalized with respect to that of peanut *AhACT11*, and data are shown as means ± standard deviation (n = 3).

To investigate the tissue-specific expression pattern of *AhGPAT9*, the relative expression of *AhGPAT9* in root, shoot, stem, mature leaf, flower, gynophore and seeds at different developmental stages were analyzed by quantitative real-time PCR. The results showed that AhGPAT9 was differentially expressed in various tissues, with the highest expression in seeds, followed by leaves and shoots, and relatively low expression in other tissues (**Figure 4**). In addition, the expression level of *AhGPAT9* showed a trend of increasing and then decreasing during seed development. These results indicate that the tissue expression of *AhGPAT9* is spatio-temporal specific, and also imply that *AhGPAT9* may play a positive role in the accumulation of substances during seed development.

**Figure 5:**
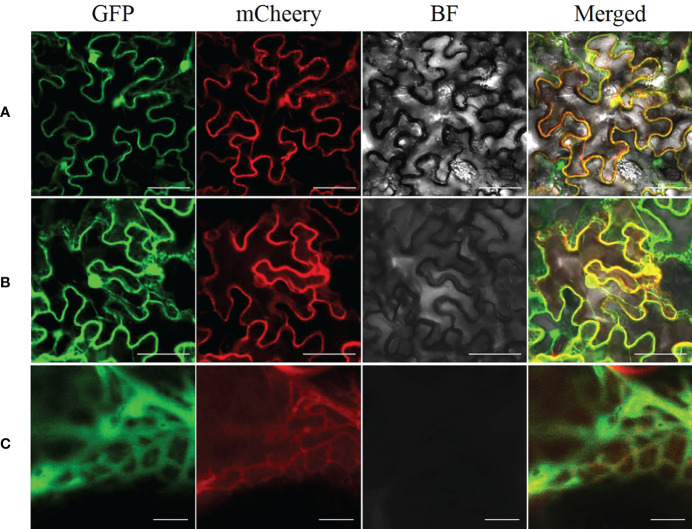
Subcellular localization of AhGPAT9 in tobacco leaves. **(A)** Representative laser-scanning images of tobacco mesophyll cells co-expressing EGFP and mCherry empty vectors (bars = 50 mm). **(B)** Representative laser-scanning images of tobacco mesophyll cells co-expressing AhGPAT9-EGFP and HDELmCherry fusion proteins (bars = 50 mm). **(C)** Enlargement of **Figure 5B** (bars = 5 mm).

We used ProtCompVersion9.0 software, an online tool for subcellular localization, to predict that AhGPAT9 was localized in the endoplasmic reticulum (ER). On that base, we expressed *AhGPAT9* fused to an *EGFP* at the C-terminal in tobacco leaves to confirm the ER localization of AhGPAT9, and used endoplasmic reticulum retention signal (HDEL) as an ER marker for co-localization (Song et al., 2021). As shown in **Figure 5**, when *AhGPAT9-EGFP* and *HDEL-mCherry* recombinants were co-expressed in tobacco mesophyll cells, the green and red fluorescence signals overlapped considerably (**Figure 5B**), and further magnification showed that the two fluorescence signals completely overlapped (**Figure 5C**), whereas unfused *EGFP* and *mCherry* was localized in cytoplasm and nucleus, as expected (**Figure 5A**). Overall, the transient expression assay turned up strong evidence that AhGPAT9 is a functional protein localized in the endoplasmic reticulum.

### *AhGPAT9* contributes to TAG accumulation and FAs alteration in seeds

To investigate the physiological functions of AhGPAT9 involved in plant oil synthesis, the coding sequence of *AhGPAT9* was introduced into a modified constitutive-expression vector for transfection using the *Arabidopsis* floral-dipping method. In this study, 37 T1 generation individual plants that overexpressed *AhGPAT9* were identified, from which 5 homozygous lines were randomly selected for tissue qRT-PCR. The results showed that the foreign gene had been successfully inserted into the genome of *Arabidopsis* and obtained stable generation (**Figure S3**).

**Figure 6:**
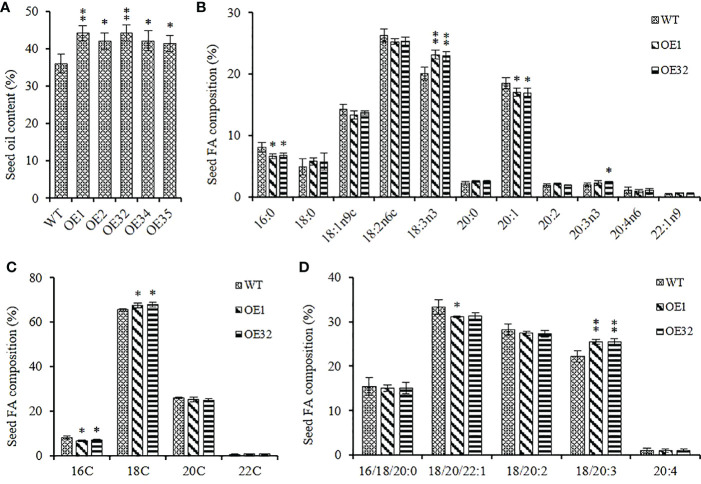
Seed oil content and composition of homozygous AhGPAT9 overexpression lines. **(A)** Oil content in seeds from wild type (WT) and AhGPAT9 overexpression lines (OE). **(B)** FA composition of seed TAG. **(C)** 16C/18C/20C/22C FAs content of seed TAG. **(D)** Saturated and unsaturated FAs content of seed TAG. Asterisks indicate significant differences from the WT: *P < 0.05; **P < 0.01 (Student’s t-test). Data are shown as means ± standard deviation (n = 5).

We extracted the seed lipid of the above transgenic lines, and found that the seed oil content of them was significantly higher than that of WT control, with an increase in range from 14.67% to 22.83%, and the mean oil content increased by about 18.73% (**Figure 6A**). Two overexpression lines of OE1 and OE32 were selected to analyze the changes in fatty acid composition for seed oil. Compared with the WT, the average content of palmitic acid (C16:0) and eicosenoic acid (C20:1) in overexpression seeds decreased by about 17.35% and 8.33%, respectively, while the average content of linolenic acid (C18:3) and eicosatrienoic acid (C20:3) increased by about 14.91% and 15.94%, respectively (**Figure 6B**). The content of 16C/18C/20C/22C FAs were then summed separately, and it was found that the content of 16C FAs in overexpression lines significantly reduced, while the content of 18C FAs significantly increased (**Figure 6C**). Similarly, the content of monounsaturated FAs significantly decreased and the content of triunsaturated FAs significantly increased in overexpression lines, while the content of saturated FAs remained stable (**Figure 6D**). These results indicate that overexpressed *AhGPAT9* not only significantly increases the oil content of transgenic seeds, but also significantly changes their FA composition ratio, suggesting that it may be preferable to utilize linolenic acid and eicosatrienoic acid as substrates during TAG assembly.

**Figure 7:**
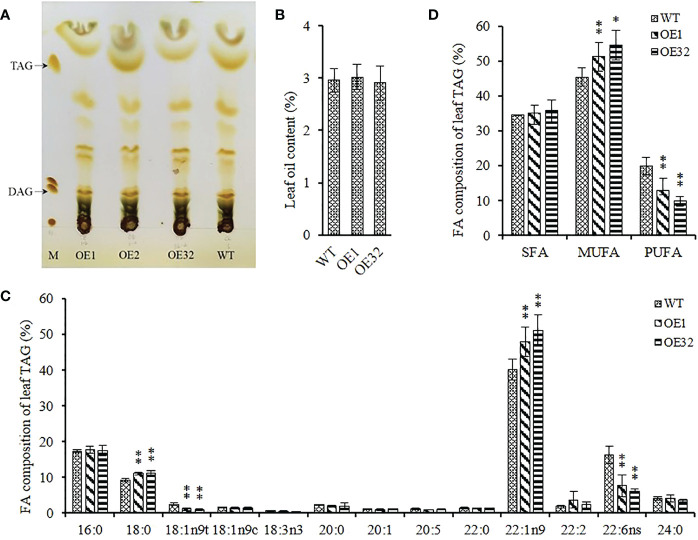
Leaf oil content and composition of homozygous AhGPAT9 overexpression lines. **(A)** Separation of lipid components from 35-40 DAG* rosette leaves by TLC. M represents a lipid marker: TAG = triacylglycerol; DAG = diacylglycerol. DAG* = days after germination. **(B)** TAG content in leaves from wild type (WT) and AhGPAT9 overexpression lines (OE). **(C)**FA composition of leaf TAG. **(D)** Saturated and unsaturated FAs content of leaf TAG. SFA = saturated fatty acid; MUFA = monounsaturated fatty acid; PUFA = polyunsaturated fatty acid. Asterisks indicate significant differences from the WT: **P < 0.01 (Student’s t-test). Data are shown as means ± standard deviation (n = 5).

Different from the mass accumulation of storage TAG in seeds, the glycerolipid in leaves are more complex and contain several polar photosynthetic membrane lipids, while TAG in mesophyll cells store in the cytosolic lipid droplets. We isolated the component of TAG from 35-40 DAG^*^ rosette leaves (**Figure 7A**), and found that there was no significant difference in TAG content between transgenic lines and WT (**Figure 7B**). Further analysis of FA composition showed that the average content of oleic acid (C18:1) and docosahexaenoic acid (C22:6) in overexpression leaves decreased by about 59.60% and 57.43%, respectively, while the average content of stearic acid (C18:0) and erucic acid (C22:1) increased by about 20.75% and 23.27%, respectively (**Figure 7C**). In addition, the content of monounsaturated FAs significantly increased and the content of polyunsaturated FAs significantly decreased in overexpressed lines, while the content of saturated FAs remained stable (**Figure 7D**). These results indicate that overexpressed *AhGPAT9* cannot significantly affect the TAG content of transgenic leaves, but does alter the FAs composition for TAG and make it inclined to recombine FAs with lower saturation, which is contrary to that in seeds.

### *AhGPAT9* affects plant growth and seed development

**Figure 8:**
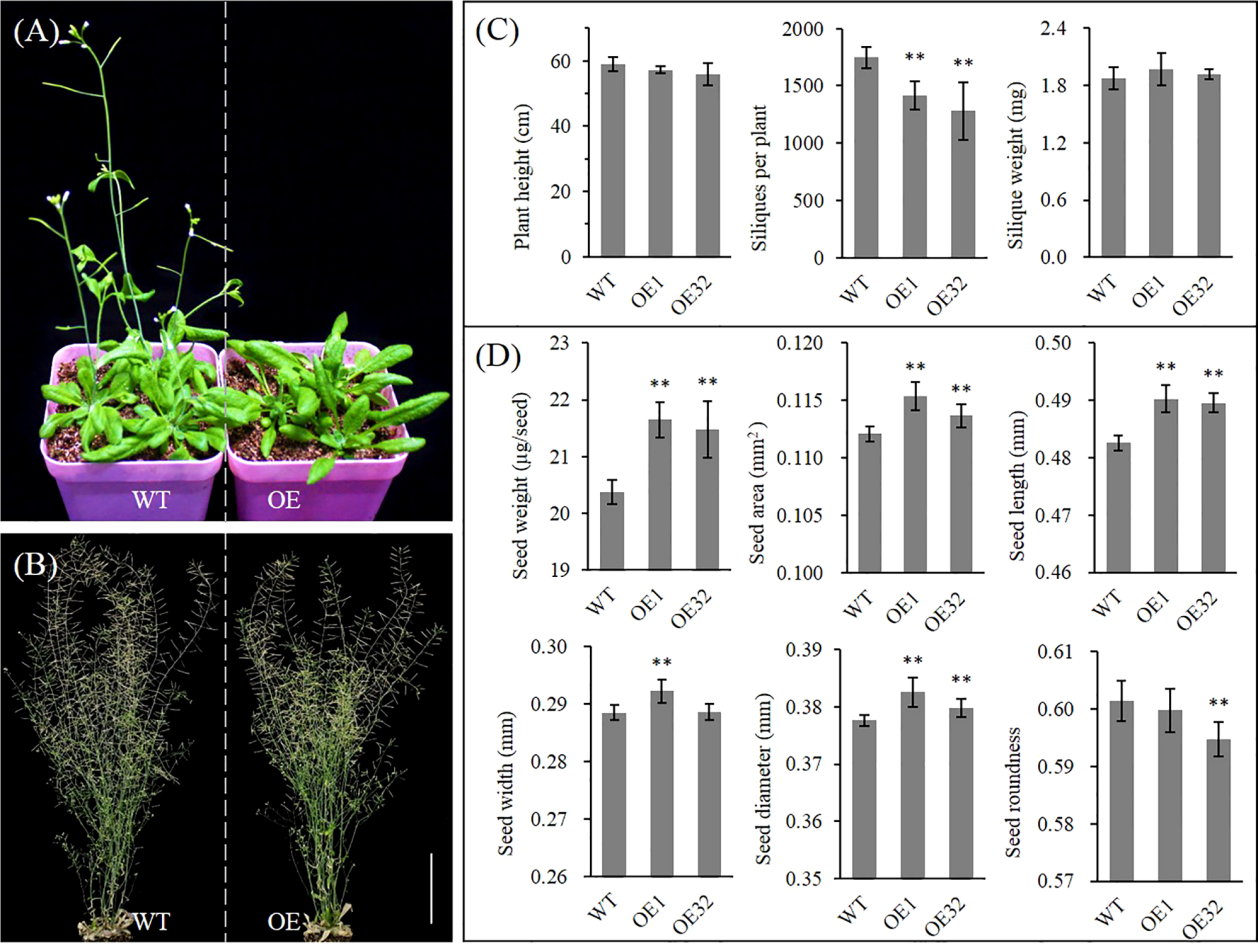
Phenotype analysis of homozygous *AhGPAT9* overexpression lines in *Arabidopsis*. **(A)** Wild type (WT) and *AhGPAT9* overexpression lines (OE) at 5-week-old under normal conditions. **(B)** Wild type (WT) and *AhGPAT9* overexpression lines (OE) at maturity under normal conditions. **(C)** Agronomic trait analysis of WT and OE lines at maturity, including plant height, siliques per plant and mean silique weight (n = 6, bars = 10 cm). **(D)** Dry seed weight and seed-shape trait analyses of WT and OE lines, including mean seed area, seed length, seed width, seed diameter and seed roundness. About 3000 seeds harvested from 12 individual plants were tested in each sample (n = 12). Asterisks indicate significant differences from the WT: **P < 0.01 (Student’s *t*-test). Data are shown as means ± standard deviation.

Under the same culture conditions, we found that overexpressed *AhGPAT9* hardly affected the seed germination and seedling growth of transgenic lines, but their bolting time of rosette was generally delayed by more than one week compared with that of the wild type control, with a corresponding delay in first-flowering (**Figures 8A** and **S4A**). The bolting rate of transgenic lines was found to variation range from 27.76% to 80.56% when grew to 5-week-old, of which four lines were significantly lower than that of wild type in the same period (**Figure S4B**). Agronomic trait analysis showed that the plant height of transgenic lines was generally shorter than that of WT at maturity, but there was no statistically significant difference. Meanwhile, the number of siliques per plant was significantly reduced, and the mean weight per silique showed an increasing trend with no statistically significant difference (**Figures 8B, C**). Compared with WT, the mean weight and area per seed for transgenic lines were significantly increased by about 5.85% and 2.15%, respectively, and other indices such as length, width, diameter and roundness of transgenic seeds also changed significantly (**Figure 8D**). These results suggest that *AhGPAT9* may be closely related to plant growth and development, especially in the reproductive growth stage, which ultimately affects the yield and quality formation of oilseeds.

## Discussion

The currently available information relevant to the potential involvement of peanut *GPATs* in glycerolipid biosynthesis is mainly derived from transcription analysis. They have spatio-temporal expression differences in various tissues, and can be induced by abiotic stress such as salt, drought and ABA treatment (Chi et al., 2015; Hao et al., 2018; Lv et al., 2020). Here, we provide genetic evidence to demonstrate that *AhGPAT9* is essential for TAG accumulation and alters the FA composition.

In this study, the homologous transcript of *GPAT9* in peanut, *AhGPAT9*, was isolated and cloned using bioinformatics methods. It was located on the 13^th^ chromosome of the peanut genome and encoded a fragment of 1131bp in nucleotide length. The polypeptide sequence encoded by *AhGPAT9* had multiple-types of phosphorylation sites and a typical PLsC domain, indicating that it belongs to the acyltransferase family. In addition, it shared 79.58% identity with the homologous polypeptide sequence in *Arabidopsis*, as well as being phylogenetically most closely related to pigeonpea (*Cajanus cajan*) and soybean (*Glycine max*) crops. Recent studies have shown that *GmGAPT9* in soybean exhibits significant acyltransferase activity by yeast genetic complementation assay, and can elevate the proportion of arachidic and erucic acids in *Arabidopsis* transgenic seeds with no changes in oil content (Liu et al., 2022). Furthermore, *in vivo* and *in vitro* experiments showed that *AtGAPT9* is highly specific for acyl-CoA and contributes to the biosynthesis of both leaf polar lipids and seed oil, as well as lipid droplet production in developing pollen grains. Loss of function results in a lethal phenotype of male and female gametophytes (Shockey et al., 2016; Singer et al., 2016). Given that GPAT9 is highly conserved throughout evolution and is largely present as one single copy in most plants, we suggest GPAT9 in peanut is very likely to encode essential housekeeping functions similar to those in *Arabidopsis* and soybean, implying the possibility that *AhGPAT9* is essential for TAG biosynthesis.

We found that *AhGPAT9* was significantly more abundantly expressed in seeds compared with other peanut tissues, with a bell-shaped expression pattern at seed developmental stage. This was consistent with the conclusion that *AhGAPT9* transcripts reached the maximum value at 42DAP (Lv et al., 2020), during which the embryo morphology was close to maturation and the seed oil accumulated rapidly. Our previous GUS fusion results indicated that *AhGPAT9* displayed the strongest activity at podding stage, especially in the developing siliques and the corresponding walking-stick embryos, which was similar to the expression pattern of *AtGAPT9* (Singer et al., 2016; Shen et al., 2022). The walking-stick embryo is in the late stage of embryonic development, during which the storage compounds are mainly accumulated to prepare for seed dormancy and germination. Taken together, the spatio-temporal expression pattern of *AhGPAT9* exhibits tissue specificity in seeds/embryos at various developmental stages, and its expression abundance is consistent with the rate of oil accumulation, suggesting that *AhGPAT9* may be a limiting factor affecting seed glycerolipid biosynthesis. The ER-bound properties of AhGPAT9 demonstrates its participation in the ER-localized glycerolipid compartmentalized biosynthesis in peanut (Gidda et al., 2009; Chapman and Ohlrogge, 2012).

In plants, TAG accumulate mainly in seeds, pericarps, leaves and flowers as neutral storage lipids (Yang and Benning, 2018). Knockdown or overexpression of *Arabidopsis* GPAT9 had been shown to affect the levels of TAG both in leaves and seeds (Shockey et al., 2016; Singer et al., 2016), which hinted at the potential functional properties of peanut homologs. Previous studies indicated that *AhGPAT9A/B* alleles indeed affect the accumulation of seed oil in peanut, and its higher oil content can be achieved not only by over-expressing *AhGPAT9*, but also may be associated with the polymorphic combination of the two alleles (Lv et al., 2020). In this study, constitutive *AhGPAT9-OE* resulted in large seed oil enhancement, while the lipid content of rosette leaves was not affected, and the TAG accumulation only accounted for about 3% of dry weight, indicating that *AhGPAT9* might be a major contributor to TAG biosynthesis in oilseeds. We further analyzed the FA composition of TAG in overexpressed seeds, and found that the proportion of linolenic acid (C18:3) and eicosatrienoic acid (C20:3) were significantly elevated compared with wild type lines, whereas palmitic acid (C16:0) and eicosenoic acid (C20:1) decreased notably, suggesting that *AhGPAT9* may display preferences for some specific PUFA substrates *in vivo*. Specially, α-linolenic acid (ALA/C18:3) is an essential omega-3 fatty acid and dietary component for human health, which can be converted into eicosapentaenoic acid (EPA) and docosahexaenoic acid (DHA) for the development of nerve cells and brain, as well as anti-allergy and anti-lipemic (Lee et al., 2018; Zhang et al., 2018). Peanut kernels are mainly rich in oleic and linoleic acids, while the content of linolenic acid is less than 1% or even undetectable (Artemis, 2001). Thus, Overexpressed *AhGPAT9* to increase linolenic acid content will be helpful to improve the fatty acid composition of peanut and expand its functional health effects. There has been a view that the substrate preferences of endogenous acyltransferases play an important role in the utilization of unusual fatty acids in transgenic oilseeds (Snyder et al., 2009). Our research helps to support the contribution of the potential role of GPAT in TAG biosynthesis and its FA recombination.

Overexpressed *AhGPAT9* delayed the bolting time of transgenic lines and reduced the siliques number, but the weight and size of progeny seeds increased significantly, speculating that *AhGPAT9* might affect florescence and seed development of plant. Combined with the contribution of *AhGPAT9* to the biosynthesis and accumulation of oil in transgenic seeds, all these results point to a positive regulatory role for *AhGPAT9* in glycerolipid metabolism, and it may run through the whole process of seed development, which has great significance for the rational design of peanut lipid traits by biotechnology in the future. Of course, peanut oil synthesis is a quantitative trait genetically controlled by multiple genes and easily affected by the environment, so it is difficult to achieve precise regulation by changing the expression of one single gene, especially given that GPAT9 catalyzes the first step in the acylation reaction of the Kennedy pathway. TAG biosynthesis needs to undergo synergistic catalysis by three acyl-assembling enzymes (Chapman and Ohlrogge, 2012; Bates et al., 2013), and the effective flux and their cumulative effects in glycerolipid metabolism need to be further explained.

## Data availability statement

The original contributions presented in the study are included in the article/**Supplementary material**. Further inquiries can be directed to the corresponding authors.

## Author contributions

YueS wrote the manuscript. YiS and YueS conceived the study and analyzed the data. YueS, YL, ML, and XZ performed experiments. YB provided valuable references and revision ideas. ZC revised the manuscript. All authors contributed to the article and approved the submitted version.

## Supplementary material

Supplementary Table 1 Primers sequences for PCR

Supplementary Figure 1 PCR amplification of peanut *AhGPAT9* gene. M represents a 2000 bp DNA marker, and the single band in right lane represents the target fragment of *AhGPAT9*.

Supplementary Figure 2 Structure analysis of peanut AhGPAT9 protein. (A) Hydrophobic prediction in ProtScale. (B) Secondary structure prediction in SOPMA. Blue = α-helix; Green = β-turn; Red = extended strand; Yellow = random coil. (C) Phosphorylation sites prediction in NetPhos (threshold > 0.5). (D) Transmembrane helices prediction in TMHMM. (E) Functional domain prediction in SMART.

Supplementary Figure 3 Expression of *AhGPAT9* in overexpression lines of *Arabidopsis*. Transcript level of *AhGPAT9* in 35-40 DAG^*^ rosette leaves and 12-15 DAF siliques of *Arabidopsis* wild type (WT) and *AhGPAT9*-overexpressed lines (OE). DAG^*^ = days after germination; DAP = days after pollination. The relative mRNA abundance was normalized with respect to that of peanut *AhACT11*, and data are shown as means ± standard deviation (n = 3).

Supplementary Figure 4 Bloting phenotype of homozygous *AhGPAT9* overexpression lines in *Arabidopsis*. (A) Wild type (WT) and *AhGPAT9* overexpression lines (OE) at 5-week-old under normal conditions. (B) Bloting rate statistics of WT and OE lines. Asterisks indicate significant differences from the WT: ***P* < 0.01 (Student’s *t*-test). Data are shown as means ± standard deviation (n = 12).
