# Supplementary material for: Characterization and functional analysis of AhGPAT9 gene involved in lipid synthesis in peanut (Arachis hypogaea L.)
Source: Front Plant Sci. 2023 Feb 10;14:1144306. doi: 10.3389/fpls.2023.1144306 (PMC9950565; doi:10.3389/fpls.2023.1144306)
Supplement: Supplementary Table 1 — Primers sequences for PCR [file Table_1.docx]

| Primer name | Forward sequence (5’-3’) | Reverse sequence (5’-3’) |
| --- | --- | --- |
| AhGPAT9-CDS | ATGATGAGGAAGACCAATCCC | TTACTTTTCTTCCAAGCGCC |
| AhGPAT9-OE | gctctaga^(XbaI)^ATGATGAGGAAGACCAATC | ggggtacc^(KpnI)^TTACTTTTCTTCCAAGCG |
| AhGPAT9-LOC | gctctaga^(XbaI)^ATGATGAGGAAGACCAATC | ggggtacc^(KpnI)^CTTTTCTTCCAAGCGC |
| AhGPAT9-RT | CGCCTGTGTGATTTGCTTGA | AACAGTAGAACCCTTGCAGGGA |
| AhACT11 | TTGGAATGGGTCAGAAGGATGC | AGTGGTGCCTCAGTAAGAAGC |
