# Supplementary figures and images for: Characterization and functional analysis of AhGPAT9 gene involved in lipid synthesis in peanut (Arachis hypogaea L.)
Source: Front Plant Sci. 2023 Feb 10;14:1144306. doi: 10.3389/fpls.2023.1144306 (PMC9950565; doi:10.3389/fpls.2023.1144306)

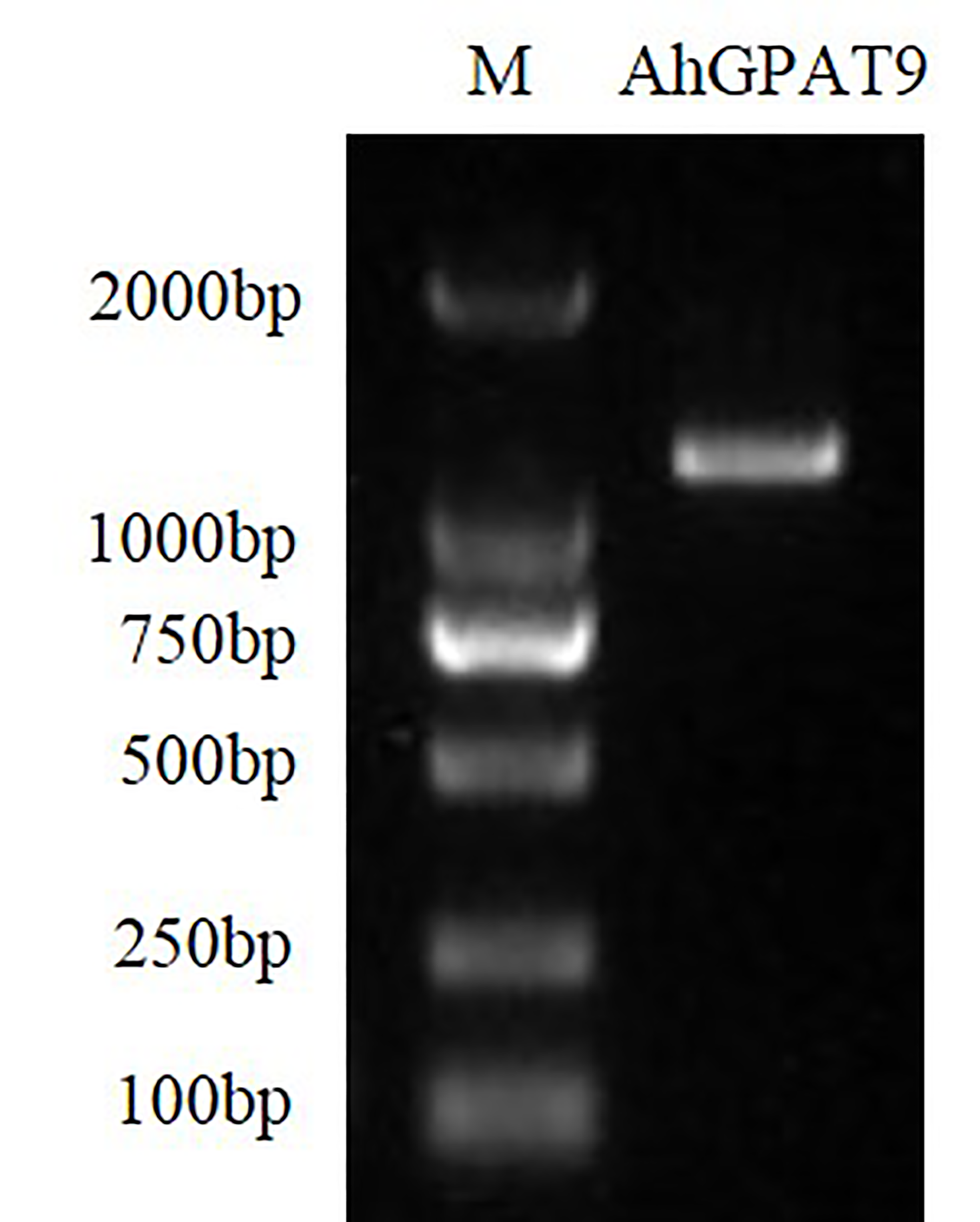

Supplement: Supplementary Figure 1 — PCR amplification of peanut AhGPAT9 gene. M represents a 2000 bp DNA marker, and the single band in right lane represents the target fragment of AhGPAT9. [file Image_1.tif]

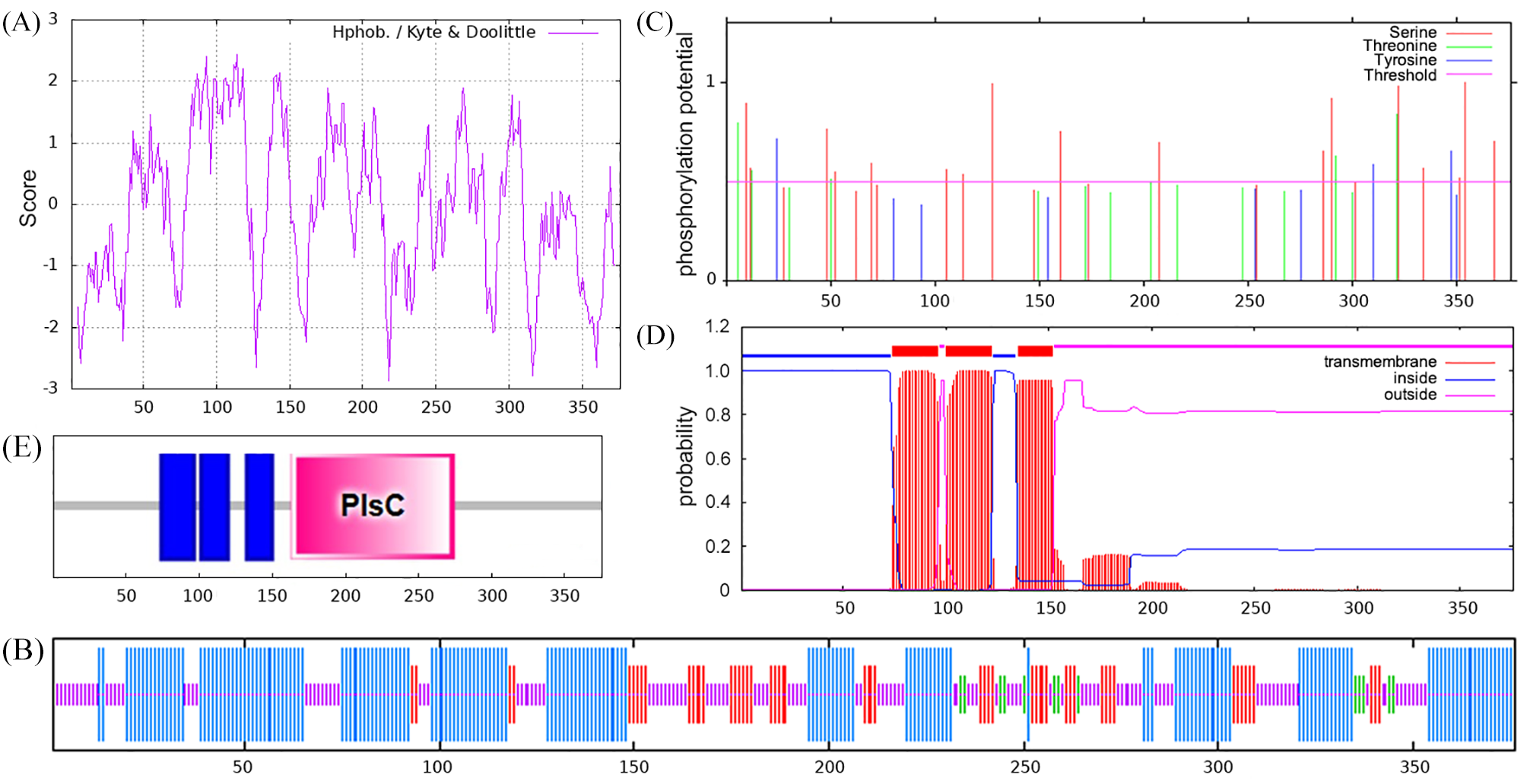

Supplement: Supplementary Figure 2 — Structure analysis of peanut AhGPAT9 protein. (A) Hydrophobic prediction in ProtScale. (B) Secondary structure prediction in SOPMA. Blue = α-helix; Green = β-turn; Red = extended strand; Yellow = random coil. (C) Phosphorylation sites prediction in NetPhos (threshold > 0.5). (D) Transmembrane helices prediction in TMHMM. (E) Functional domain prediction in SMART. [file Image_2.tif]

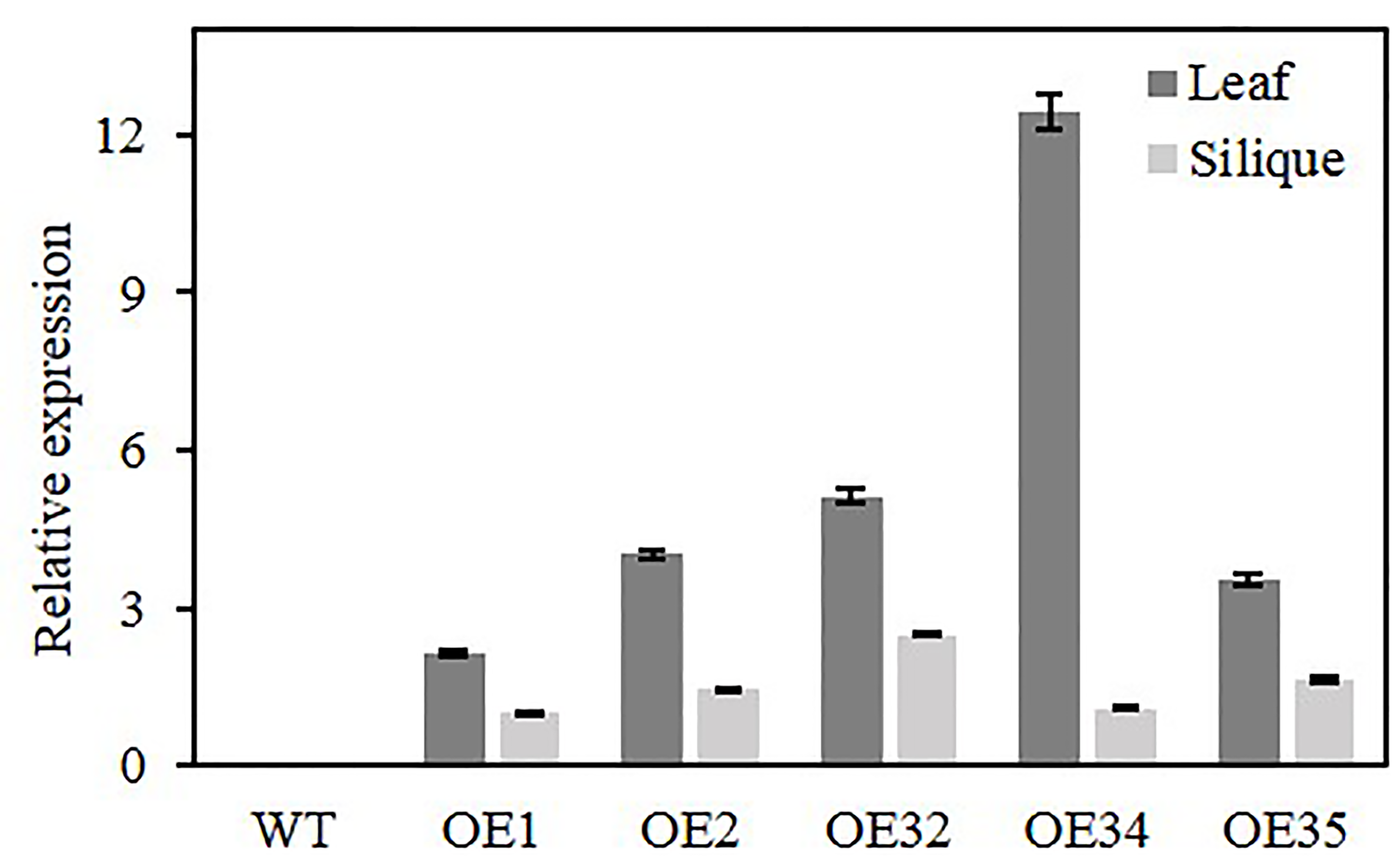

Supplement: Supplementary Figure 3 — Expression of AhGPAT9 in overexpression lines of Arabidopsis. Transcript level of AhGPAT9 in 35-40 DAG* rosette leaves and 12-15 DAF siliques of Arabidopsis wild type (WT) and AhGPAT9-overexpressed lines (OE). DAG* = days after germination; DAP = days after pollination. The relative mRNA abundance was normalized with respect to that of peanut AhACT11, and data are shown as means ± standard deviation (n = 3). [file Image_3.tif]

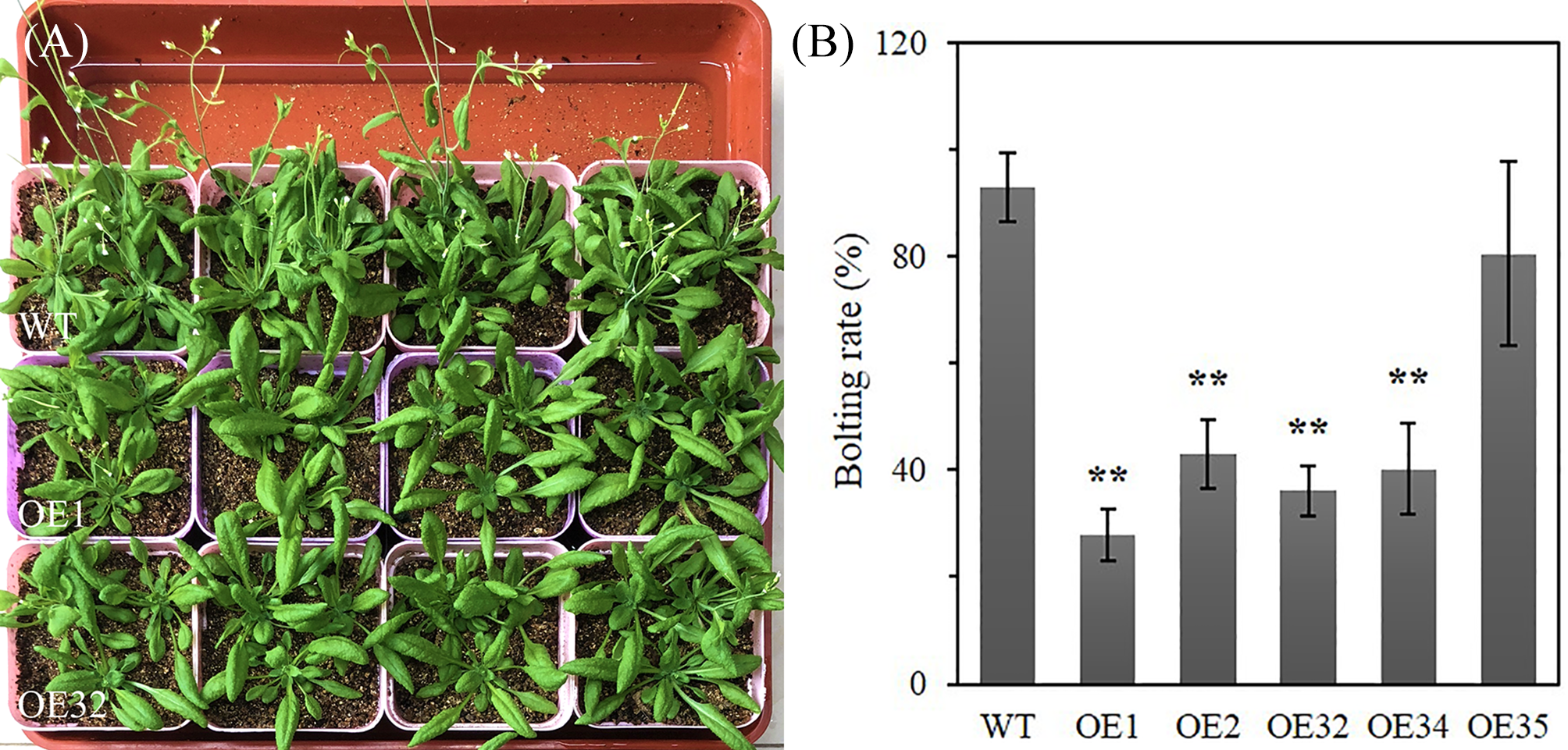

Supplement: Supplementary Figure 4 — Bloting phenotype of homozygous AhGPAT9 overexpression lines in Arabidopsis. (A) Wild type (WT) and AhGPAT9 overexpression lines (OE) at 5-week-old under normal conditions. (B) Bloting rate statistics of WT and OE lines. Asterisks indicate significant differences from the WT: **P < 0.01 (Student’s t-test). Data are shown as means ± standard deviation (n = 12). [file Image_4.tif]
